# 

**Published:** 1914-07

**Authors:** 


### INDEX TO VOLUME XXX.

ORIGINAL ARTICLES— Page.

A Contribution to the Study of Joint-Bodies from Within Present in Articulations Otherwise Apparently Normal. By Aime Paul Heinecke, M. D., Chicago, Ill ....... 399, 495
A Model Practice Act. By H. Sheridan Baketel, A. M., M. D., New York .. 6
An Indiana Child's Creed ....... 211
A Report of the Surgeons' Congress. By Dr. Joe Gilbert, Austin, Texas .. 109
Artificial Pneumo-Thorax. By A. G. Shortle, M. D., Albuquerque, N. M. 299, 447
Auto-intoxication. By J. W. McLaughlin, M. D., Austin, Texas ....... 149
Belladonna. By E. S. Goodhue ....... 154
Chorea and Its Treatment. By Dr. G. Wilse Robinson, Kansas City, Mo .. 500
Conditions Modifying Results of Operations. By Dr. J. M. Inge, Denton, Texas ....... 408
Crotalin in the Use of Epilepsy. By Dr. J. Gordon Brydson, Bastrop, Texas 509
Experimental Rhus Poisoning. By L. B. Bibb, M. D., Austin, Texas ....... 162
Important Factors to be Considered in the Diagnosis and Treatment of Carcinomata of the Breast. By Dr. F. C. Floeckinger, Taylor, Texas ... 99
Malnutrition and Its Treatment. By Dr. Emile Bruner, New York ....... 207
Observations on Lacerated and Contused Wounds. By L. Sexton, B. S., M. D., Tulane ....... 51
Pellagra, Its Symptoms and Treatment. By L. P. Tenny, Troup, Texas .. 449
Procidentia or Prolapsus of Uteri. By P. D. Spohn, M. D ....... 199
Progress and Advancement of Medical Education in Texas. By W. L. Crosthwait, Secretary Texas State Board of Medical Examiners ....... 55
Rabies. By R. H. Bibb, M. D ....... 155
Reminiscences. By M. A. Taylor, M. D ....... 3
Report of Four Cases of What Appeared to be Tuberculosis Meningitis with Apparent Permanent Arrestment. By Chas. C. Browning, M. D., Los Angeles, Cal ....... 249
Some Phases of Malaria. By R. H. Crockett, M. D., Hutto, Texas ....... 544
Some Recent Observations of Pyorrhea Alveolaris, or Rigg's Disease. By C. C. Bass, M. D., New Orleans, La ....... 261
Some Surgical Complications Following Influenza. By W. L. Crosthwait .. 249
Spirit and Drug Taking Among Doctors. By T. D. Crothers, M. D., Hartford, Conn. ....... 111
Stomach Troubles. By F. C. Floeckinger, M. D., Taylor, Texas ....... 503
Surgery of the Ovaries and Tubes and the Indications Thereof. By Dr. R. L. Hall, Italy, Texas ....... 410
The New Independence: Made in America. By G. Henri Bogart, M. D., Paris, Ill. ....... 547
The Physicians of Hawaii. By Dr. E. S. Goodhue ....... 511
The Texas Health Train ....... 112
The Use of Obstetric Forceps. By T. J. Turpin, M. D., Corpus Christi, Texas ....... 352
Transplantation of the Ovary to Avoid Dysmenorrhea and Premature Menopause. By Z. T. Scott, M. D., Austin, Texas ....... 1

DEPARTMENT OF EUGENICS— Page.

A Court Decision. By Dr. Malone Duggan.......... 15
A Criminologist's View of the Divorce Question. By Dr. Malone Duggan.. 166
An Opportune Suggestion. By Dr. Malone Duggan.......... 216
A Warning to Eugenists. By Dr. Malone Duggan.......... 327
Consecration of the Mental Health and Care of the Insane. By Dr. Malone Duggan.......... 168
Dr. White on Insanity.......... 218
Eugenics and Ethics. By Dr. Malone Duggan.......... 65
Eugenic Legislation. By Dr. Malone Duggan.......... 519
Eugenic Notes. By Dr. Malone Duggan.......... 213
Father. Exchange.......... 325
Father and Son. Ella Wheeler Wilcox.......... 21
Feeble-mindedness.......... 459
Free Women and the Birth Rate. By Dr. Malone Duggan.......... 517
Galston's Resume of Eugenics. By Dr. Malone Duggan.......... 417
Honor and Eugenics. By Dr. Malone Duggan.......... 516
Illegitimate. By Dr. Malone Duggan.......... 120
Is the Sex Instinct the Strongest? By Dr. Malone Duggan.......... 119
Malthusianism and the War. By Dr. Malone Duggan.......... 217
Mendel's Law Demonstrated. By Dr. Malone Duggan.......... 325
Morality and Sex. By Dr. Malone Duggan.......... 165
Neo-malthusianism and Eugenics. By Dr. Malone Duggan.......... 456
Obsessed. By Dr. Malone Duggan.......... 13
Organic and Social Heredity. By Dr. Malone Duggan.......... 415
Parental Influence and Race Betterment. By Dr. Malone Duggan.......... 70
Present Status of Sex Education. By Dr. Malone Duggan.......... 267
Psychopathic Hospitals. By Dr. Malone Duggan.......... 329
Sex Hygiene. By Dr. Malone Duggan.......... 11
Social Hygiene. By Dr. Malone Duggan.......... 418
Some Recent Contributions to Eugenics. By Dr. Malone Duggan.......... 115
Symptoms of Social Disorganization. By Theo. Y. Hull.......... 561
Texas Congress of Mothers. By Dr. Malone Duggan.......... 270
Texas Promoting Social Hygiene. By Dr. Malone Duggan.......... 11
Texas State Congress of Charities and Correction. By Dr. Malone Duggan. 271
The American Social Hygiene Association. By Dr. Malone Duggan.......... 523
The Moral Sense. By Dr. Malone Duggan.......... 416
War, Psychology and Eugenics. By Dr. Malone Duggan.......... 118
What Then? By Dr. Malone Duggan.......... 67
Why Not? By Dr. Malone Duggan.......... 12
Women and Chivalry. By Dr. Malone Duggan.......... 68
World's Purity Association. By Dr. Malone Duggan.......... 417

EDITORIAL DEPARTMENT—

Alcohol and the Medical Profession.......... 223
A Letter to Subscribers.......... 274
Antisepsis and Asepsis. By Geo. H. Lee, M. D.......... 336
A Plea for the Kind Reception of the Young Doctor.......... 130
Apposing Child Labor.......... 28
A Widening Scope.......... 28
City Sanitation.......... 73

Page.
Corporis Humani Fabrica of Andreas Versalis........................ 427
Delayed Letters of Condolence........................................ 32
Doctors as Leaders...................................................... 223
Dr. Geo. H. Moody, President 1915-16................................ 565
Dr. Bacon Saunders Honored........................................... 338
Dr. J. M. Inge, President-elect........................................ 567
Eating for Efficiency. M. M. Carrick, M. D........................ 527
Especially Concerning the Country and Small Town General Practitioner. By C. H. Hughes, St. Louis........................................ 335
Health First Instead of Safety First. By M. M. Carrick, M. D......... 72
Ill-health from New Houses............................................. 29
Is There a Conspiracy of Silence..................................... 174
Lessons from the Bubonic Plague...................................... 76
Letters Concerning the Package Library.............................. 340
Newspaper Campaigns for Public Health.............................. 30
Operating to Cure Criminal Impulses.................................. 74
Our Ensign ................................................................ 128
Pellagra and Beri-beri. By L. B. Bibb, Austin, Texas................ 526
Prejudice Against the Doctor. By M. M. Carrick...................... 224
Prevalence of Pellagra.................................................. 29
Some of Our Plans for the Future...................................... 562
State Association Meeting a Great Success.......................... 568
Thank You ................................................................. 174
The Coming Southern Medical Association. By Edward H. Cary, M. D.... 528
The Cost of Sickness in Texas. By M. M. Carrick, M. D............... 426
The Higher Law in Medicine. By T. D. Crothers...................... 470
The Insurance Forum. By M. B. Grace, M. D......................... 475
The Medical Package Library.......................................... 468
The Necessity of Centralization of Power for Protecting Public Health. By Frank Paschal, M. D.............................................. 26
The New Independence: Made in America. By G. Henri Bogart........ 473
The New State Health Officer.......................................... 375
The Physician's Prayer.................................................. 273
The Texas Surgical Society............................................. 469
The War and Sexual Morality. By G. Henri Bogart.................... 223
Why We Should Buy Goods Made in the United States. By J. W. Lambert, M. D.............................................................. 372
Work in the New Field. By T. D. Crothers, M. D...................... 424

ABSTRACTS AND SELECTIONS..................140, 185, 236, 385, 430, 434

ITEMS OF GENERAL INTEREST............35, 83, 136, 175, 228, 288, 342, 381, 435, 479, 488, 529, 531,........................................ 572

PUBLISHER'S DEPARTMENT....48, 96, 145, 192, 224, 294, 348, 394, 444, 490

BOOK REVIEWS.........................................190, 242, 390, 528, 536

### The Woman's Department

**CONDUCTED BY MRS. F. E. DANIEL**

COLLABORATORS

**Dr. J. S. Abbott, Dairy and Food Commissioner of Texas; Miss Eleanor Brackenridge, President of the Equal Franchise Society, San Antonio; Dr. G. Henri Bogart, Associate Editor, Utah Medical Journal, Paris, Ill.; Mrs. Isabella Charles Davis, Second Vice President National Order of "King's Daughters," New Jersey; Mrs. Jno. S. Turner, Recording Secretary, Texas Congress of Mothers; Dr. Marvin B. Grace, Seguin; Dr. Margaret Holliday, Women's Physician, University of Texas, Austin; Dr. John S. Turner, President State Medical Association; Dr. Ralph Steiner, State Health Officer; Dr. L. H. Sackett, Instructor in the Philosophy of Education, University of Texas; Dr. F. S. White, Supt. S. W. Texas Lunatic Asylum, San Antonio, Texas; Mrs. M. C. Kersh, Dallas, Texas.**

VITAL

FACTS

EVERY

WOMAN

SHOULD

KNOW.

*Teach me the way to a strong body, a pure mind, a noble character.*

*"Ignorance is not innocence."*

A LIST OF NEW SUBSCRIBERS.—We are glad to announce that the following doctors have been added to the list of the "Red Back" family since we last went to press. We bid them welcome, and hope that they may "live long and prosper," and always be loyal to the TEXAS MEDICAL JOURNAL:

Dr. D. S. Alverson, Houston, Texas; Dr. H. C. Morrow, Austin, Texas; Dr. B. H. Farr, El Paso, Texas; Dr. Carlos M. Riveroll, San Antonio, Texas; Dr. W. Olvera Zuniga, El Paso, Texas; Dr. C. F. Fowler, Galveston, Texas; Dr. J. Lozano Gonzales, Eagle Pass, Texas; Dr. E. V. Fulton, Denton, Texas; Dr. H. Payne, Poteet, Texas; Dr. Eusebio Guajardo, Victoria, Texas; Dr. E. E. Collins, Premont, Texas; Dr. A. Collado, Brownsville, Texas; Dr. Adolfo Salinas-Puga, Laredo, Texas; Dr. G. A. Deason, Dallas, Texas; Dr. T. J. Crowe, Dallas, Texas; Dr. Geo. L. Carlisle, Dallas, Texas; Dr. J. S. Cooper, Houston, Texas; Dr. W. O. Padgett, Dallas, Texas; Drs. Denson, Queen & Van Zant, Cameron, Texas; Dr. Orville T. Bundy, Hutto, Texas; Dr. Edmond Doak, Taylor, Texas; Dr. B. R. Davis, Laredo, Texas; Dr. P. Martinez, Corpus Christi, Texas; Dr. D. R. Aves, Seabrook, Texas; Dr. C. G. Stricklin, Gustine, Texas; Dr. J. R. Beall, New York.

---

*Doctor:*

You are cordially invited to tell the readers of the TEXAS MEDICAL JOURNAL about any of your cases that may be interesting and that you feel will prove helpful to a brother physician.

Whenever you are impressed by a condition that appeals strongly to you, remember that there are hundreds of doctors who would be glad to know just how you handled the case and with what measure of success.

It does not take long to write up a case, and it may prove most valuable to the profession at large. In this way you will help the profession of the State to keep well abreast of the times and to set a higher standard for them from year to year.

A Texas physician is much more interested in knowing what his fellow practitioner is doing and accomplishing than he is in knowing what some big somebody—or other—on the other side of the Ocean is dreaming about.

Let us hear from you.

